# Die Rolle von kardialen Biomarkern in der perioperativen Risikoevaluation von nichtkardiochirurgischen Patienten – eine Zusammenfassung der ESAIC-Leitlinie 2023

**DOI:** 10.1007/s00101-023-01363-4

**Published:** 2023-12-08

**Authors:** René M’Pembele, Sebastian Roth, Giovanna Lurati Buse

**Affiliations:** 1 Klinik für Anästhesiologie, Universitätsklinikum Düsseldorf, Heinrich-Heine-Universität, Moorenstr. 5, 40225 Düsseldorf, Deutschland; 2 CARID (Cardiovascular Research Institute Düsseldorf), Universitätsklinikum Düsseldorf, Heinrich Heine Universität, Düsseldorf, Deutschland

**Keywords:** Risikostratifizierung, Troponin, BNP, Prognose, Myokardschaden, Risk stratification, Troponin, BNP, Prognosis, Myocardial injury

## Abstract

**Hintergrund:**

Die ESAIC-Leitlinie aus dem Jahr 2023 beleuchtet den klinischen Wert von kardialem Troponin (cTn) und B‑Typ natriuretischen Peptiden (BNP) zur Risikoevaluation in nichtkardiochirurgischen Patienten.

**Ziele der Arbeit:**

Zusammenfassung der Empfehlungen der neuen ESAIC-Leitlinie.

**Material und Methoden:**

Die Evidenz für die Empfehlungen der Leitlinie wurde aus Studien extrahiert, die den perioperativen Nutzen von cTn und BNP für die Anwendungsbereiche der Prognoseabschätzung, Risikoprädiktion und Therapieoptimierung untersuchten. Für die Erstellung des Empfehlungsgrads wurden zusätzlich 12 relevante Endpunkte und das Risiko-Nutzen-Verhältnis der systematischen Messung der Biomarker mitberücksichtigt.

**Ergebnisse:**

Es konnten 115 Studien als Grundlage für die Leitlinienempfehlungen identifiziert werden. Die verfügbare Evidenz variierte stark zwischen den 12 verschiedenen Endpunkten. Zusätzlich zeigte sich ein Evidenzgefälle für die einzelnen Anwendungsbereiche der Biomarker. Es wurden schwache Empfehlungen für die präoperative, postoperative und sequenzielle Messung von cTn und die präoperative Messung von BNP zur Prognoseabschätzung abgegeben. Für die Risikoprädiktion wurde ebenfalls eine schwache Empfehlung für die sequenzielle und postoperative Messung von cTn sowie präoperative Messung von BNP abgegeben. Die Evidenz von kardialen Biomarkern zur Therapieoptimierung war unzureichend, sodass ihr Nutzen unklar blieb und keine Empfehlung abgegeben werden konnte.

**Diskussion:**

Kardiale Troponine und BNP können bei nichtkardiochirurgischen Patienten für die Prognoseabschätzung und Risikoprädiktion für ausgewählte Endpunkte verwendet werden. Therapieentscheidungen sollten nicht aufgrund der Erhöhung dieser Biomarker getroffen werden.

## Abstract (en)

**Background:**

The recently published ESAIC guidelines highlight the clinical value of cardiac troponins (cTn) and B‑type natriuretic peptides (BNP) for risk assessment in patients undergoing noncardiac surgery.

**Objective:**

Summary of the ESAIC guideline recommendations.

**Material and methods:**

The evidence for the recommendations was extracted from studies that investigated the perioperative role of cTn and BNP as prognostic factors, for risk prediction and for therapeutic guidance. To collate this evidence 12 relevant endpoints as well as risk benefit analyses of systematic screening were considered to issue the strength of the recommendations.

**Results:**

The body of evidence for these guidelines was based on 115 studies. The evidence varied significantly across the 12 predefined endpoints. Additionally, there was a gradient in evidence for the use of cTn and BNP as prognostic factors, for risk prediction and for therapeutic guidance. The guidelines issue a weak recommendation for the use of preoperative, postoperative and combined measurement of cTn as well as for preoperative BNP measurement to assess the prognosis. For risk prediction a weak recommendation was formulated for combined and postoperative cTn and preoperative BNP measurements. No recommendation could be given for the evidence on biomarkers as data were very limited.

**Conclusion:**

Both cTn and BNP can be used as prognostic factors or to predict the risk for selected endpoints. Therapeutic interventions should not be guided by cardiac biomarker levels.

## Hintergrund

Weltweit nimmt die Anzahl an nichtkardiochirurgischen Operationen in den letzten Jahrzehnten stetig zu [21, 22]. In Europa müssen schätzungsweise zwischen 5000 und 5500 nichtkardiochirurgische Eingriffe pro 100.000 Einwohner durchgeführt werden, um der Krankheitslast der Bevölkerung gerecht zu werden [17]. Ein zunehmendes Patientenalter sowie eine Zunahme komplexer Eingriffe führen zu einem steigenden perioperativen Risiko [14]. Nach nichtkardiochirurgischen Eingriffen sind kardiovaskuläre Komplikationen mit einer erhöhten Morbidität und Mortalität vergesellschaftet, und der Tod nach nichtkardiochirurgischer Operation gilt als dritthäufigste Todesursache in Industrieländern [6, 19]*.*

Auf der einen Seite ist die präoperative Risikoevaluation von Patienten vor nichtkardiochirurgischen Eingriffen essenziell, um das Risiko für postoperative Komplikationen abzuschätzen und somit eine valide Patientenaufklärung vornehmen zu können. Andererseits kann die präoperative Risikoevaluation für den Versuch genutzt werden, das Outcome der Patienten durch gezielte klinische Interventionen zu beeinflussen.

Neben klinischen Risiko-Scores, die aus dem Komorbiditätsprofil der Patienten abgeleitet sind, werden auch kardiale Biomarker zur Risikoevaluation verwendet [3, 9, 15, 20]. Die in diesem Zusammenhang am häufigsten untersuchten Biomarker sind kardiales Troponin (cTn) und B‑Typ natriuretische Peptide (BNP). Der prognostische Wert von erhöhten Plasmakonzentrationen dieser Biomarker für das Auftreten verschiedener postoperativer Komplikationen konnte in Beobachtungsstudien mehrfach gezeigt werden [1, 2, 7, 11, 16, 24].

Aktuelle Empfehlungen der europäischen Fachgesellschaften für Kardiologie und Anästhesiologie (ESC und ESAIC) zur Erhebung von kardialen Biomarkern für die präoperative Risikoevaluation divergieren in der Empfehlungsstärke und der Population, in der eine präoperative Erhebung kardialer Biomarker als sinnvoll betrachtet wird [4, 8]. Vor allem die im Jahre 2022 aktualisierte Leitlinie der ESC empfiehlt eine routinemäßige prä- und postoperative Messung von cTn und BNP bei einer sehr viel breiteren Patientenpopulation und mit höherem Empfehlungsgrad, verglichen mit der ESAIC-Leitlinie von 2018 [4, 8]. Diese Diskrepanzen führen zu einer unzureichenden Handlungssicherheit im klinischen Umgang mit der Erhebung kardialer Biomarker.

Um den Nutzen von kardialen Biomarkern im klinischen Alltag transparenter zu machen, wurden im Juni 2023 die neue ESAIC-Leitlinie zur perioperativen Risikoevaluation durch kardiale Biomarker veröffentlicht [10]. Diese Übersichtsarbeit beleuchtet wichtige Eckpunkte dieser Leitlinie und fasst ihre Empfehlungen zusammen.

## Welche kardialen Biomarker wurden in der neuen ESAIC-Leitlinie evaluiert?

Die in der klinischen Routine meist genutzten kardialen Biomarker sind, wie bereits erwähnt, die cTn und die BNP. Aufgrund des klaren Bezugs zur klinischen Praxis, den bereits bestehenden Empfehlungen von ESC und ESAIC und der Vielzahl an bereits vorhandenen Studien fokussieren sich die ESAIC-Empfehlungen auf diese beiden kardialen Biomarkergruppen [10].

## Anwendungsgebiete von Biomarkern zur Risikoevaluation und relevante Messzeitpunkte

In der neuen ESAIC-Leitlinie unterscheiden die Autoren zwischen 3 verschiedenen Anwendungsbereichen von kardialen Biomarkern zur perioperativen Risikoevaluation von nichtkardiochirurgischen Patienten:

1. Prognoseabschätzung,
2. Risikoprädiktion,
3. biomarkergestützte Therapieoptimierung.

Die Prognoseabschätzung beschreibt den Zusammenhang zwischen erhöhten Plasmaspiegeln des Biomarkers und dem Risiko für das Auftreten eines Endpunktes. Bei der Risikoprädiktion ist neben der reinen Assoziation mit einem Endpunkt auch der Aspekt relevant, wie präzise der Biomarker Patienten mit erhöhtem Risiko identifizieren kann. Im Bereich der biomarkergestützten Therapieoptimierung ist v. a. von Interesse, ob eine durch den Biomarker angezeigte Intervention das Outcome von identifizierten Hochrisikopatienten verbessern kann. Diese Unterscheidung der einzelnen Anwendungsgebiete wurde in den bereits existierenden Leitlinien zur präoperativen Risikoabschätzung bislang nur unzureichend berücksichtigt. Da die Anwendungsgebiete jedoch sehr heterogen sind und Ergebnisse aus einem Bereich nicht zwangsläufig auf die anderen beiden übertragbar sind, wurden aus den vorhandenen Daten getrennte Empfehlungen für die Anwendungsbereiche abgeleitet. Kardiale Biomarker können prä- sowie postoperativ, aber auch durch eine sequenzielle Messung (prä- und postoperativ) erhoben werden und damit zur Prognoseabschätzung, Risikoprädiktion und Therapieoptimierung beitragen. Basierend auf der Datenlage wurden in der neuen ESAIC-Leitlinie Empfehlungen für die einzelne prä- oder postoperative sowie für die kombinierte prä- und postoperative Bestimmung formuliert [10].

## Identifikation relevanter Endpunkte und Methodik

Die Autoren der Leitlinie definierten in einem modifiziertem Delphi-Prozess 12 klinisch relevante Endpunkte für Patienten, die sich einem nichtkardiochirurgischen Eingriff unterziehen. Hierbei wurden u. a. Endpunkte wie die Mortalität oder postoperative Komplikationen innerhalb der ersten 30 Tage oder eines Jahres nach der Operation, aber auch patientenzentrierte Endpunkte zusammengefasst. Eine detaillierte Darstellung der einzelnen Endpunkte findet sich in Tab. 1 [10].

**Table 1:** 

Relevante Studienendpunkte nach nichtkardiochirurgischen Eingriffen
Gesamtmortalität bis zu 30 Tagen nach Operation
Gesamtmortalität bis zu 1 Jahr nach Operation
Kardiovaskulärer Tod bis zu 30 Tagen nach Operation
Tod oder Myokardinfarkt bis zu 30 Tagen nach Operation
Tod oder Myokardinfarkt bis zu 1 Jahr nach Operation
Schwere kardiale Komplikationen bis zu 30 Tagen nach Operation
Schwere kardiale Komplikationen bis zu 1 Jahr nach Operation
Kardiale Komplikationen (jeder Schweregrad) bis zu 30 Tagen nach Operation
Myokardschaden bis zu 30 Tagen nach Operation („Non-occlusive“-Troponinerhöhung)
Gesamtkomplikationen bis zu 30 Tagen nach Operation
Behinderung bis zu 90 Tagen nach Operation
Lebensqualität bis zu 90 Tagen nach Operation

Gemäß dem „*Grading of Recommendations Assessment Development and Evaluation*“ (GRADE) Framework, spielten bei der Verfassung der Empfehlungen, neben der Evaluation der Studienqualität, auch Aspekte der Risiko-Nutzen-Abwägung der Biomarkerbestimmung eine Rolle. In diesem Zusammenhang wurden die klinische Relevanz des Problems, die Größe der erwarteten positiven und negativen Effekte durch die Biomarkermessung, die benötigten Ressourcen und damit verbundene Einflüsse auf das Gesundheitswesen sowie die Durchführbarkeit und Akzeptanz durch Vertreter der involvierten Fachbereiche (Anästhesiologie, Kardiologie, Chirurgie, Labormedizin, Patientenvertretung) als relevante Faktoren in die Entscheidungsfindung miteinbezogen.

## Allgemeine Ergebnisse

Während der Literaturrecherche konnten aus 25.359 Suchergebnissen 115 relevante Studien extrahiert werden. Die Analyse der verfügbaren Studien hat ergeben, dass sowohl Quantität als auch Qualität der Evidenz für die verschiedenen definierten Endpunkte für cTn, aber auch für BNP stark variieren. So gab es beispielsweise eine sehr hohe Evidenz für cTn (prä-, postoperativ und sequenzielle Messung) als prognostischen Faktor für die 30-Tages-Mortalität, wohingegen die Evidenz für den Einfluss von cTn auf das Auftreten von kardialen und nichtkardialen Komplikationen innerhalb von 30 Tagen, abhängig vom Messzeitpunkt, zwischen niedrig und sehr niedrig variierte. Zusätzlich ergab sich auch ein deutliches Evidenzgefälle zwischen den einzelnen Anwendungsgebieten für kardiale Biomarker. Während für die Prognoseabschätzung viele Studien existierten, gab es für die biomarkergestützte Therapieoptimierung kaum Daten (Tab. 2). Zusätzlich gab es auch deutliche Unklarheiten im Risiko-Nutzen-Verhältnis der systematischen perioperativen Erhebung kardialer Biomarker.

**Table 2:** 

	Studien zur Prognoseabschätzung	Studien zur Risikostratifizierung	Studien zur Therapieoptimierung
*cTn, präoperativ*	16 Studien	7 Studien	Keine
*cTn, sequenziell*	12 Studien	3 Studien	2 Studien
*cTn, postoperativ*	29 Studien	12 Studien	4 Studien
*BNP, präoperativ*	56 Studien	16 Studien	Keine
*BNP, postoperativ*	9 Studien	1 Studien	Keine

*BNP* B-Typ natriuretische Peptide, *cTn* kardiale Troponine

## Empfehlungen zur Prognoseabschätzung durch kardiale Biomarker

**Figure 1:**
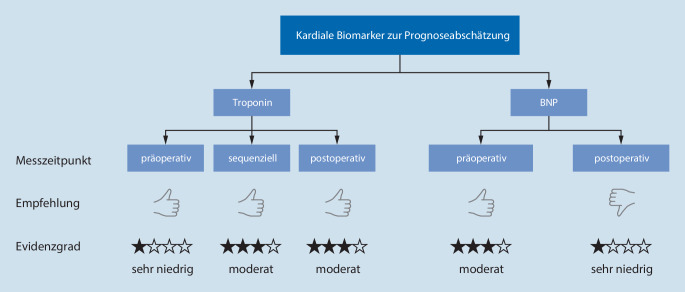

Die ESAIC-Leitlinie empfiehlt mit einem schwachen Empfehlungsgrad, dass prä- und postoperative sowie sequenzielle cTn-Messungen zur Prognoseabschätzung durchgeführt werden können. Auch präoperative BNP-Messungen können zur Prognoseabschätzung verwendet werden. Die Autoren schlagen insbesondere vor, dass diese Messungen routinemäßig durchgeführt werden können, um Patienten über das Risiko für postoperative Komplikationen zu informieren. Der schwache Empfehlungsgrad resultiert hauptsächlich aus der moderat bis schwachen gepoolten Evidenz aus allen Endpunkten. Hervorzuheben ist, dass für den Endpunkt der 30-Tages-Mortalität für alle cTn-Messpunkte eine gute Datenlage vorhanden ist. Für präoperative BNP-Messungen existiert wiederum eine gute Datenlage für die Endpunkte kardiovaskulärer Komplikationen und Tod oder Myokardinfarkt innerhalb von 30 Tagen postoperativ. Für postoperative BNP-Werte zur Prognoseabschätzung liegt eine sehr eingeschränkte Evidenz vor, sodass der Routineeinsatz nicht empfohlen wird ([10]; Abb. 1).

## Empfehlungen zur Risikoprädiktion durch kardiale Biomarker

**Figure 2:**
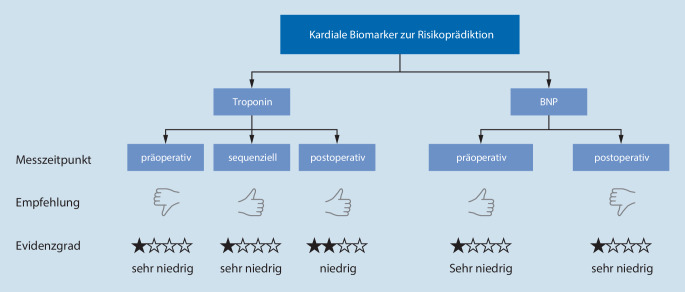

Für die Risikoprädiktion durch kardiale Biomarker empfiehlt die ESAIC-Leitlinie, dass sequenzielle und postoperative cTn- sowie präoperative BNP-Messungen durchgeführt werden können. Die Empfehlung ist ebenfalls schwach aufgrund der schwachen Evidenz, und es wird darauf hingewiesen, dass Risikoprädiktion nur für bestimmte Endpunkte sinnvoll erscheint, um die Performance von klinischen Prädiktionsmodellen und Risiko-Scores zu verbessern. Hierbei gibt es eine gute Evidenz für den prädiktiven Wert von postoperativen cTn-Messungen und der 30-Tages-Mortalität sowie eine moderate Evidenz für den prädiktiven Wert von sequenzieller cTn- und präoperativer BNP-Messung und dem Auftreten von kardiovaskulären Komplikationen. Die routinemäßige Bestimmung von präoperativen cTn- und postoperativen BNP-Werten wird nicht empfohlen ([10]; Abb. 2).

## Empfehlungen zur biomarkergestützten Therapieoptimierung

**Figure 3:**
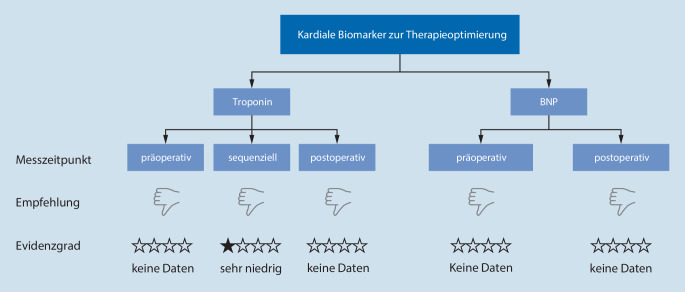

Die aktuelle Datenlage zur biomarkergestützten Therapieoptimierung ist sehr spärlich. Daher können die Autoren der Leitlinie keine Empfehlung für die routinemäßige Messung von prä- und postoperativem cTn und BNP abgeben, um diagnostische oder interventionelle Therapieentscheidungen zu stützen. Nichtsdestotrotz führt die Leitlinie an, dass die cTn-Dynamik aus sequenziellen Messungen nützlich sein könnte, um zwischen akuter postoperativer Myokardschädigung und chronischer cTn-Erhöhung zu unterscheiden, die dann ggf. eine Therapiekonsequenz nach sich ziehen könnte. Zusätzlich wird empfohlen, dass Zentren, die bereits standardmäßig sequenziell cTn bestimmen, diese Messungen in einen wissenschaftlichen Kontext einbetten sollten, um die zukünftige Datenlage zu verbessern ([10]; Abb. 3).

## Diskussion

Die ESAIC-Leitlinie zur Rolle von kardialen Biomarkern in der perioperativen Risikoevaluation von nichtkardiochirurgischen Patienten 2023 kommt nach einer systematischen Analyse der vorhandenen Daten zu dem Schluss, dass nur schwache Empfehlungen für die routinemäßige Nutzung von kardialen Biomarkern in den Bereichen der Prognoseabschätzung, der Risikoprädiktion und der biomarkergestützten Therapieoptimierung abgegeben werden können [10].

Diese Empfehlungen stehen auf den ersten Blick im Kontrast mit den von der ESC abgegebenen starken Empfehlungen, die für Risikopatienten eine präoperative sowie postoperative cTn-Überwachung über 48 h fordert. Auch für die präoperative Messung von BNP wird in der ESC-Leitlinie von 2022 eine Empfehlung zugunsten von BNP-Bestimmungen in diesem Patientenkollektiv abgegeben [17]. Beide Leitlinien erkennen den prognostischen Wert von erhöhten kardialen Biomarkern und die Relevanz des perioperativen Myokardschadens an. Dennoch stellt sich die Frage, warum die Empfehlungen voneinander divergieren.

Der Kernunterschied besteht in der Methodologie der beiden Leitlinien. Durch die vorherige Festlegung von 12 klinisch relevanten Endpunkten konnte die ESAIC-Leitlinie sehr sensitiv Evidenzlücken für einzelne dieser Endpunkte identifizieren. Zusätzlich wurde durch die Unterscheidung in die Anwendungsbereiche für kardiale Biomarker die Aussagekraft für Prognose, Risikostratifizierung und biomarkergestützte Therapieoptimierung für den klinischen Alltag präzisiert. Hierdurch wird die Bedeutung einer erhöhten Biomarkerkonzentration im klinischen Alltag greifbarer und kann auch im Rahmen der Arzt-Patient-Kommunikation zur Information von Patienten genutzt werden. Diese methodologische Vorgehensweise hat systematisch die Evidenzlücken aufgezeigt, die in den nächsten Jahren durch klinische Forschungsprojekte geschlossen werden müssen [10].

Besonders im Bereich der biomarkergestützten Therapieoptimierung bleiben Fragen offen. Es ist fraglich ob durch Biomarkererhöhung getriggerte Interventionen (beispielsweise präoperative Echokardiographie oder medikamentöse Optimierung) oder aber auch durch die routinemäßige Messung kardialer Biomarker selbst das Outcome der Patienten beeinflusst wird. Die unklare Datenlage bezüglich geeigneter Therapien des perioperativen Myokardschadens und der damit verbundenen Unklarheit über das Risiko-Nutzen-Verhältnis von systematischem Screening von Hochrisikopatienten mittels kardialer Biomarker erschwert die Abgabe von starken Empfehlungen zur routinemäßigen Messung dieser Marker in der neuen ESAIC-Leitlinie [18].

## Expertenmeinung zur klinischen Anwendung der Leitlinie

Aus dieser Zusammenfassung der ESAIC-Leitlinie wird ersichtlich, dass eine Empfehlung der routinemäßigen Erhebung von kardialen Biomarkern nicht durch die entsprechende Evidenz gestützt werden kann. In einigen Kliniken werden jedoch bereits kardiale Biomarker prä- sowie postoperativ teils routinemäßig, teils jedoch auch zufällig bestimmt. In diesem Kontext fragen sich die Behandelnden, welche therapeutischen Interventionen bei Erhöhung dieser Werte sinnvoll sein könnten. Bei präoperativ erhöhten cTn- und BNP-Werten vor elektiven Eingriffen macht es Sinn, die Patienten über das erhöhte perioperative Risiko bezüglich postoperativer (kardialer) Komplikationen und 30-Tages-Mortalität aufzuklären. Dies könnte im Rahmen einer zusätzlichen Risikoaufklärung erfolgen und könnte einen Einfluss auf die Patientenentscheidung haben, einen elektiven Eingriff durchführen zu lassen. Zusätzlich liegt es nahe, diese Patienten mit erhöhtem präoperativ identifiziertem Risiko intra- und postoperativ engmaschiger zu überwachen. Denkbare Interventionen wären hier Hypotensionsvermeidungsstrategien, invasive Blutdruckmessung und die postoperative (Intensiv‑)Überwachung sowie das weitere postoperative Monitoring kardialer Biomarker. Obwohl diese Interventionen sinnvoll erscheinen, gibt es aktuell keine Hinweise, dass sie das Outcome dieser Patienten nachhaltig verbessern. Der zusätzliche Nutzen der präoperativen (Stress‑)Echokardiografie ist in diesem Kontext ebenfalls unklar. Eine präoperative (elektive) Koronarintervention scheint in diesem Kontext nicht zielführend zu sein, da Studien keine Reduktion des Auftretens früher postoperativer Komplikationen nachweisen konnten [12, 23]. Bei postoperativ erhöhtem cTn gibt es jedoch Hinweise, dass zu einem späterem postoperativem Zeitpunkt (> 7 Tage postoperativ) eine sekundärprophylaktische Antikoagulation mit Dabigatran sinnvoll sein könnte, um das Risiko für vaskuläre Komplikationen über die nächsten 2 Jahre zu reduzieren [5]. Diese Evidenz betrachten wir jedoch als nicht suffizient, um diese Therapie zu empfehlen. Sie könnte jedoch in ausgewählten Einzelfällen erwogen werden, nachdem das Nutzen-Risiko-Verhältnis sorgfältig abgewogen wurde. Bei Patienten mit postoperativ erhöhtem cTn sollte ein multidisziplinärer Ansatz mit Einbeziehung der Kardiologie angestrebt werden [13].

## Zusammenfassung und Fazit für die Praxis

Zusammenfassend können cTn und BNP bei nichtkardiochirurgischen Patienten für die Prognoseabschätzung und Risikoprädiktion zum Auftreten einiger Endpunkte verwendet werden. Da es für die biomarkergestützte Therapieoptimierung keine überzeugenden Daten gibt, sollten keine Therapieentscheidungen von cTn- oder BNP-Konzentrationen abgeleitet werden. Zu guter Letzt kann die routinemäßige Messung der kardialen Biomarker aufgrund der nur schwachen Empfehlungen aktuell nur für wissenschaftliche Zwecke befürwortet werden. In den kommenden Jahren müssen die aufgedeckten Evidenzlücken geschlossen werden, um die Rolle von kardialen Biomarkern in der perioperativen Risikoevaluation abschließend bewerten zu können.

## Weiterführende Links (ESAIC-Leitlinie – kardiale Biomarker)

- https://journals.lww.com/ejanaesthesiology/Fulltext/9900/ESAIC_focused_guideline_for_the_use_of_cardiac.98.aspx
- https://www.esaic.org/uploads/2023/07/esaic_biomarker_guidelines_digital.pdf?_gl=1*1sp9thl*_up*MQ..*_ga10049/MS/1253/*MTIyMzcyOTAxNS4xNjk3NjEzMTUx*_ga_RGL0Z35SXM*MTY5NzYxMzE1MS4xLjAuMTY5NzYxMzE1MS4wLjAuMA
